# Clinicopathological features of fibrosarcomatous dermatofibrosarcoma protuberans and the construction of a back-propagation neural network recognition model

**DOI:** 10.1186/s13023-021-01698-4

**Published:** 2021-01-26

**Authors:** Yanan Li, Jiaqi Liang, Xuewen Xu, Xian Jiang, Chuan Wang, Siyuan Chen, Bo Xiang, Yi Ji

**Affiliations:** 1 Division of Oncology, Department of Pediatric Surgery, West China Hospital of Sichuan University, #37 Guo-Xue-Xiang, Chengdu, 610041 China; 2 Shaanxi Zhongtian Rocket Technology Co, Ltd, Xi’an, 710025 China; 3 Department of Burns and Plastic Surgery, West China Hospital of Sichuan University, Chengdu, 610041 China; 4 Department of Dermatology, West China Hospital of Sichuan University, Chengdu, 610041 China; 5 Pediatric Intensive Care Unit, Department of Critical Care Medicine, West China Hospital of Sichuan University, Chengdu, 610041 China

**Keywords:** Dermatofibrosarcoma protuberans, Fibrosarcomatous, Clinicopathological features, BP neural network

## Abstract

**Background:**

Fibrosarcomatous dermatofibrosarcoma protuberans (FS-DFSP) is a form of tumor progression of dermatofibrosarcoma protuberans (DFSP) with an increased risk of metastasis and recurrence. Few studies have compared the clinicopathological features of FS-DFSP and conventional DFSP (C-DFSP).

**Objectives:**

To better understand the epidemiological and clinicopathological characteristics of FS-DFSP.

**Methods:**

We conducted a cohort study of 221 patients diagnosed with DFSP and built a recognition model with a back-propagation (BP) neural network for FS-DFSP.

**Results:**

Twenty-six patients with FS-DFSP and 195 patients with C-DFSP were included. There were no differences between FS-DFSP and C-DFSP regarding age at presentation, age at diagnosis, sex, size at diagnosis, size at presentation, and tumor growth. The negative ratio of CD34 in FS-DFSP (11.5%) was significantly lower than that in C-DFSP (5.1%) (*P* = 0.005). The average Ki-67 index of FS-DFSP (18.1%) cases was significantly higher than that of C-DFSP (8.1%) cases (*P* < 0.001). The classification accuracy of the BP neural network model training samples was 100%. The correct rates of classification and misdiagnosis were 84.1% and 15.9%.

**Conclusions:**

The clinical manifestations of FS-DFSP and C-DFSP are similar but have large differences in immunohistochemistry. The classification accuracy and feasibility of the BP neural network model are high in FS-DFSP.

## Introduction

Dermatofibrosarcoma protuberans (DFSP) is a rare and low-grade cutaneous soft tissue sarcoma with intermediate malignancy [1]. It is estimated that the incidence is approximately 0.8 to 5 cases per million per year [2, 3]. DFSP is most frequently observed in the Black race [4]. The male-to-female ratio is nearly 1:1 [5]. DFSP is usually diagnosed in adults in their 20 s, 30 s and 40 s [6, 7] and mostly occurs on the trunk [8]. The local recurrence rate of DFSP is high, but the metastasis rate is low (approximately 2–5%) [3, 9, 10]. Penner first described metastatic DFSP with fibrosarcomatous (FS) areas in 1951 [11]. The frequency of FS change according to histopathology may be 5% to 15% of DFSP cases, with a high rate of local and distant metastasis [12, 13]. It has been suggested that FS change might be a risk factor for local recurrence [14–17].

An artificial neural network (ANN) is an intelligent system that learns how the brain processes information by imitating the human nervous system. ANNs can make correct predictions of unknown data by learning and testing known data, and they do this by mathematically and physically abstracting and mimicking the structure and function of the human brain [18]. A back-propagation (BP) neural network is a kind of multilayer feedforward network that uses the error back-propagation algorithm. It has been reported that approximately 90% of neural networks are based on the BP algorithm, which has been widely used in disease recognition and diagnosis [19, 20].

Currently, few studies have reported the differences of conventional DFSP (C-DFSP, without fibrosarcomatous change) and FS-DFSP in the clinical features. In order to deeply understand the clinical characteristics of DFSP, we conducted a retrospective cohort study to evaluate the clinical characteristics of FS-DFSP and C-DFSP and build a recognition model with a BP neural network.

## Methods

**Fig. 1:**
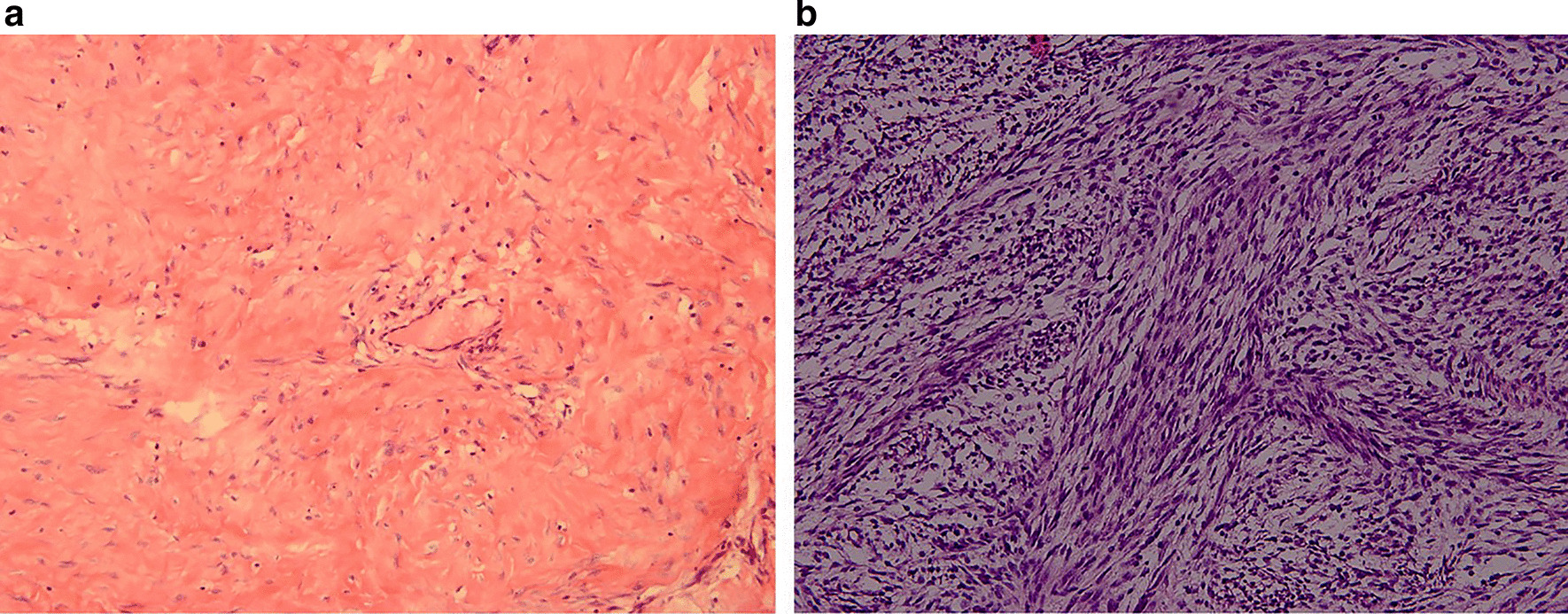
Histopathological examination. **a** DFSP without fibrosarcomatous change showing spindle cells (H&E, original magnification × 200). **b** DFSP with fibrosarcomatous change showing a fascicular growth pattern (H&E, original magnification × 200)

This study was a retrospective analysis of 221 patients with FS-DFSP (26) and C-DFSP (195) between 2010 and 2019 at the West China Hospital of Sichuan University. Approval was obtained from the West China Hospital of Sichuan University institutional review board. We got written informed consent from all patients or their parents when necessary. The diagnosis of DFSP was based on histological data. Clinical information, including sex, age at presentation, age at the time of first diagnosis, tumor size, location, histopathological findings, follow-up and outcome, was obtained. According to histopathology, we divided DFSP into two types: C-DFSP (Fig. 1a) and FS-DFSP (Fig. 1b) [21].

Proportions were calculated for categorical variables, and means were calculated for continuous variables. The Pearson chi-square test and Fisher’s exact test were used to analyze categorical variables. Continuous variables were analyzed by using Student’s *t*-test. SPSS 25.0 for Windows (SPSS, Inc., Chicago, IL, USA) was used for statistical analyses. P values less than 0.05 indicated statistically significant results.

**Fig. 2:**
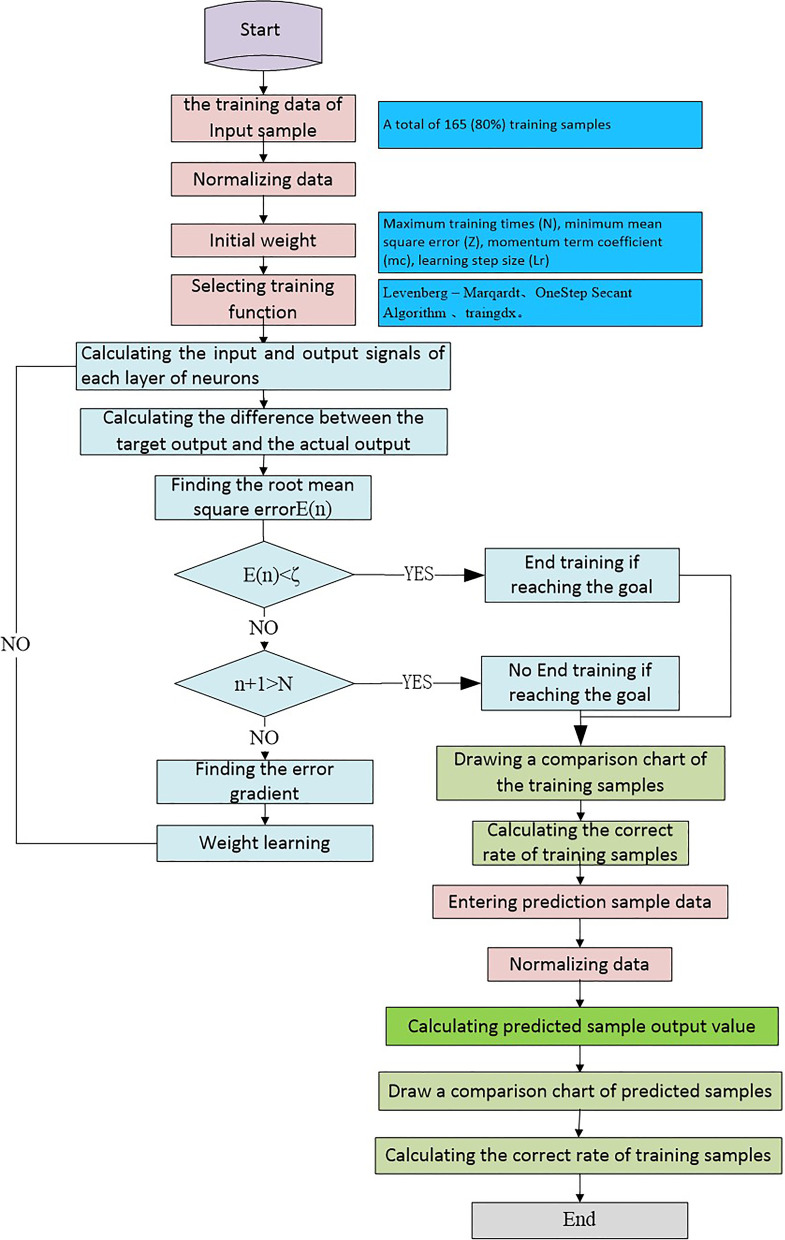
The topological structure of the BP neural network

The Levenberg–Marquardt algorithm was provided in the MATLAB neural network to build a recognition model with a BP neural network. The number of input nodes for this study is 10: X0 = sex, X1 = age at presentation, X2 = age at diagnosis, X3 = the interval of diagnosis, X4 = location, X5 = size at presentation, X6 = size at diagnosis, X7 = tumor growth, X8 = annual tumor growth, and X9 = growth type. The number of hidden neural nodes is 3. The number of output layer nodes is 1, corresponding to 1 for FS-DFSP and 0 for C-DFSP. The topological structure of the BP neural network is shown in Fig. 2.

## Results

### Patient characteristics

**Fig. 3:**
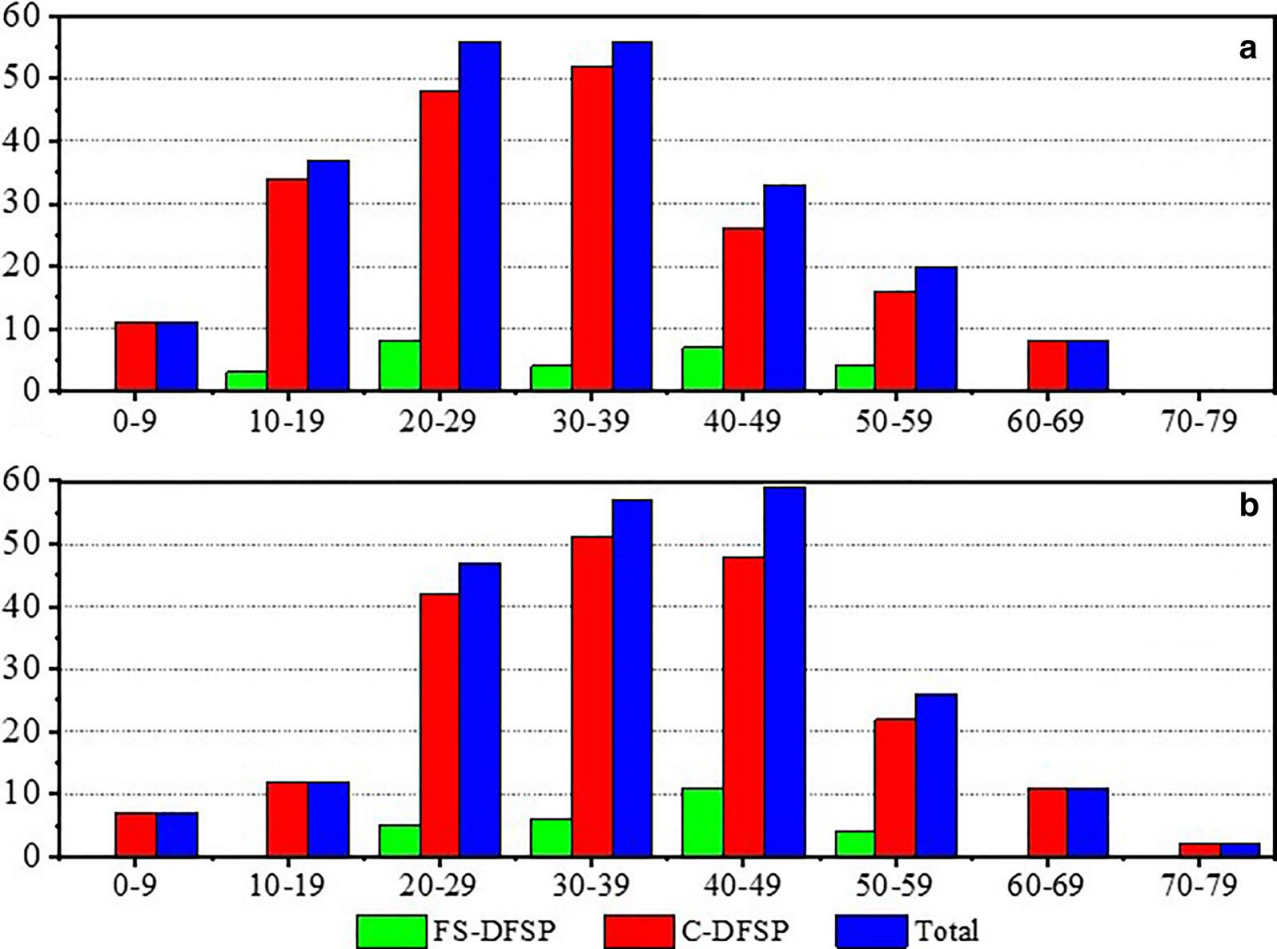
Age distribution at presentation and first diagnosis in patients with FS-DFSP and C-DFSP (Y-axis: number of patients, X-axis: years). **a** Age at presentation; **b** age at first diagnosis

In total, 221 patients with a diagnosis of DFSP were included. All patients were Chinese. Table 1 presents the main clinical features of DFSP in this study. In our sample, there was a predominance of male patients, with a male-to-female ratio of 1:0.75. The peak incidence of DFSP at diagnosis was observed in patients in their 30 s to 50 s (Fig. 3a). The peak incidence of DFSP at presentation (the tumor was first noticed by the patient) was observed in patients whose ages ranged from 12 years to the sixth decade of life (Fig. 3b). DFSP mostly occurred on the chest (26.7%). The "presumed" causes of the tumors were trauma (8.1%) and unknown (91.9%). In 43.9% of patients, the tumors were first indolent after discovery for a certain period of time but later grew rapidly. The median time to rapid enlargement (time from discovery of the tumor to rapid enlargement of the tumor) was 3.0 years. In 37.6% of patients the tumor size was persistently stable, and in 19.8% growth increased gradually. In most cases, patients (86.0%) presented with a painless plaque, and only 31 patients (14.0%) had painful masses.

**Table 1:** Clinical features of conventional and fibrosarcomatous DFSP

Variables	C-DFSP	FS-DFSP	Total	*P* value
	*n* = 195	*n* = 26	*n* = 221	
Age at presentation (y)				
Mean ± SD	30.7 ± 14.4	35.4 ± 12.7	31.24 ± 14.23	0.091
Median (range)	30.0 (0–66.0)	36.5 (12–57.0)	30.25 (0.0–66.0)	
Age at diagnosis (y)				
Mean ± SD	37.3 ± 14.5	40.5 ± 10.3	37.69 ± 14.06	0.168
Median (range)	37.0 (0.3–76.3)	43.1 (20.0–59.0)	37.5 (0.3–76.3)	
The interval of diagnosis (y)				
Mean ± SD	6.5 ± 6.9	5.1 ± 6.5	6.3 ± 6.9	0.302
Median (range)	4.3 (0–44.0)	1.8 (0.3–20.7)	4.0 (0–44.0)	
Sex (male/female)	108/87	18/8	126/95	0.18
Size at presentation (cm)				
Mean ± SD	1.1 ± 1.0	1.0 ± 0.7	1.07 ± 1.05	0.818
Median (range)	1.0 (0.2–10.0)	1.0 (0.1–3.0)	1.0 (0.1–10.0)	
Size at diagnose				
Mean ± SD	2.7 ± 2.0	3.1 ± 1.7	2.8 ± 2.0	0.362
Median (range)	2.0 (0.5–20.0)	3.0 (1.0–8.0)	2.50 (0.5–20.0)	
Tumor growth				
Mean ± SD	1.7 ± 1.8	2.0 ± 1.7	0.9 ± 0.3	0.337
Median (range)	1.0 (0–17.0)	1.7 (0–7.0)	1.5 (0–17.0)	
Annual tumor growth				
Mean ± SD	0.9 ± 2.9	1.8 ± 2.4	0.97 ± 2.84	0.090
Median (range)	0.3 (0–37.5)	0.7 (0–9.3)	0.3 (0–37.5)	
Location				
Head-face-neck	21 (10.8%)	5 (19.2%)	26 (11.8%)	0.272
Shoulder	11(5.6%)	2(7.7%)	13 (5.9%)	
Chest	51 (26.2%)	8 (30.8%)	59 (26.7%)	
Abdomen	49 (25.1%)	3 (11.5%)	52 (23.5%)	
Posterior thighs	40 (20.5%)	8(30.8%)	48 (21.7%)	
Upper extremity	11 (5.6%)	0 (0)	11 (5.0%)	
Lower extremity	12 (6.2%)	0 (0)	12 (5.4%)	
Growth type				
Indolence	29 (14.9%)	1 (3.8%)	30 (13.6%)	< 0.001
Gradually increasing	90 (46.2. %)	4 (15.4%)	94 (42.5%)	
Rapid enlargement	76 (38.9%)	21 (80.8%)	97 (43.9%)	
Time to rapid enlargement (y)				
Mean ± SD	5.3 ± 6.5	0.9 ± 1.1	4.4 ± 6.0	0.003
Median (range)	4.0 (0.1–41.0)	0.6 (0–5.0)	3.0 (0–41.0)	
Cause				
Trauma	18	0	18	0.140
Unknown	178 (91.3%)	26(100%)	204 (92.3%)	
Pain	29	2	31	
Metastasis	0	1	1	0.118
Recurrence	4	7	11	
Follow-up				
Mean ± SD	4.8 ± 2.2	4.9 ± 1.7	4.8 ± 2.1	0.696
Median (range)	4.6 (1.8–16.6)	4.6 (1.9–8.6)	4.6 (1.8–16.6)	

C-DFSP, conventional dermatofibrosarcoma protuberans; Time to rapid enlargement, time from discovery of the tumor to progressive enlargement of the tumor; FS-DFSP, Dermatofibrosarcoma protuberans with fibrosarcomatous change; y, years

### Comparison of clinical features between conventional and fibrosarcomatous DFSP

C-DFSP mostly occurred on the chest (26.2%), whereas FS-DFSP mostly occurred on the chest (11.8%) and posterior thighs (11.8%). Lung metastasis was found in only 1 FS-DFSP case. No differences in sex, age at presentation, age at the time of first diagnosis, interval between initial presentation and diagnostic confirmation, tumor size at the time of presentation, tumor size at the time of diagnosis, tumor growth, annual tumor growth or location were observed between C-DFSP and FS-DFSP. The annual tumor growth of FS-DFSP was significantly higher than that of C-DFSP, but there was no significant difference (*P* = 0.090).

### Immunohistochemistry

CD34 staining was positive in 88.5% of FS-DFSP cases, whereas CD34 staining was positive in 99.5% of C-DFSP cases (*P* = 0.005) (Table 2). There were no significant differences between the two groups regarding the standings of CD10, SMA or S100 (*P* > 0.05). P53 staining was negative in 24 FS-DFSP cases and 195 C-DFSP cases (*P* = 0.015). The Ki-67 average index was significantly higher in FS-DFSP than in C-DFSP (*P* < 0.001).

**Table 2:** Immunohistochemistry of conventional and fibrosarcomatous DFSP

	NC-DFSP (195)	FS-DFSP (26)	*P* value
CD34			
Negative	1	3	0.005
Positive	194	23	
CD10			
Negative	27	2	–
Positive	24	24	
Unknown	144	0	
SMA			
Negative	193	26	1.000
Positive	2	0	
Desmin			
Negative	194	26	–
Positive	0	0	
S100			
Negative	194	26	1.000
Positive	1	0	
P53			
Negative	195	24	0.015
Positive	0	2	
EMA			
Negative	195	26	–
Positive	0	0	
Ki-67	8.1 ± 4.7	18.1 ± 12.2	< 0.001

### The results of the BP neural network model

**Fig. 4:**
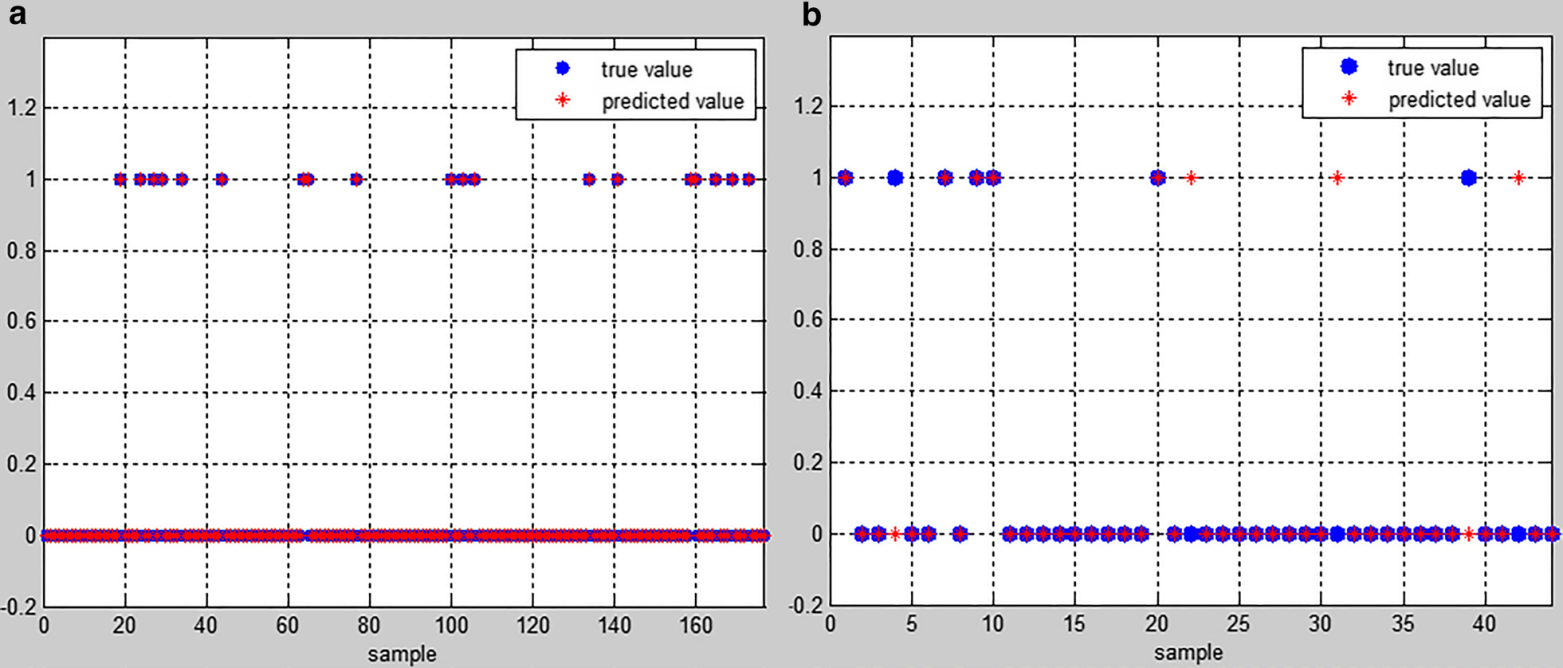
The results of the BP neural network model: **a** the result of the training set; **b** the result of the test set

The Levenberg–Marquardt algorithm can provide numerical solutions that minimize the number of nonlinearities (local minimums) with the fastest convergence speed (average 30 times). The number of hidden layers was 10, the number of trainings was 31, and the training target was 0.01. The training sample classification accuracy was 100%. The training sample misdiagnosis rate was 0 (Fig. 4a). In FS-DFSP, the test sample classification correct rate was 88.64%, and the test sample misdiagnosis rate was 11.36%. When training 31 times, the mean square error was 0.01, which reached the target value (Fig. 4b).

## Discussion

In the current study, a large number of patients were assessed the clinical features of C-DFSP and FS-DFSP. Our results indicate no significant difference between patients with C-DFSP and FS-DFSP in terms of sex, age at presentation, age at the time of first diagnosis, interval from initial presentation to diagnostic confirmation, tumor size at the time of presentation, tumor size at the time of diagnosis, tumor growth, or annual tumor growth. Interestingly, compared with that of recurrent C-DFSP, the annual tumor growth of FS-DFSP was higher, although there was no significant difference.

Recent studies have revealed that the frequency of FS according to histopathology may be approximately 5% to 15% of all DFSP cases [12, 13]. Similar to previous studies, we found that FS-DFSP represented 11.7% of all DFSP cases. Connelly et al. reported that the median age of FS-DFSP patients was significantly higher than that of C-DFSP patients [22]. In the current study, we found that the median age of patients with FS-DFSP was only slightly higher than that of patients with C-DFSP. Many studies have reported that DFSP is generally diagnosed between the ages of 20 and 40 years. However, few studies have indicated the age at presentation of DFSP [6, 7, 21]. We found that there were some differences in the age at presentation of DFSP between the two groups. In our study, the peak incidence of FS-DFSP at presentation was observed in patients in their 20 s, 30 s, 40 s and 50 s, whereas the peak incidence of C-DFSP at presentation was observed in patients aged 12 years to the 6th decade of life.

DFSP can occur anywhere in the body. We found that FS-DFSP mostly occurred on the chest and posterior thighs, whereas C-DFSP mostly occurred on the chest. Currently, the correlation between DFSP incidence and sex remains unclear. Bowne et al. reported that the male-to-female ratio was nearly 1:1 [5]. Other studies reported a slight predominance of female patients [4, 23]. In the current study, however, we observed that there was a predominance of male patients in FS-DFSP and C-DFSP. Correlations with prior trauma, surgical or burn scars, which had been reported in approximately 10% of DFSP cases, were unclear [24, 25]. In our series, trauma induced DFSP in 8.1% of patients.

Clinically, DFSP often presents as an indolent tumor [26]. In the current study, we found that some lesions can be indolent, whereas others can grow slowly or show rapid enlargement after a period of indolence. Interestingly, FS-DFSP had a significantly shorter time from indolence to rapid enlargement. FS changes have not been reported in children with DFSP [27–30]. Interestingly, the tumors of two patients with FS-DFSP presented in childhood. The age of one patient was 12 years, and that of the other was 15 years. There is evidence suggesting that FS-DFSP may be an evolution of C-DFSP. Previous studies have demonstrated that P53 and MDM2 are overexpressed in FS-DFSP. In addition, activation of Akt/mTOR, STAT3, ERK and PD-L1 may be related to the development or progression of DFSP [21, 31, 32].

Previously, the wide local excision (WLE) was the gold standard treatment for DFSP, with a recurrence rate ranging from 0 to 41% [33]. Recently, Mohs micrographic surgery (MMS) has been proven to be an alternative to WLE that assesses 100% of the margins with maximum tissue conservation. Many studies comparing the recurrence rate of WLE and MMS for the treatment of DFSP have shown that the recurrence rate after MMS ranges from 0 to 6.7% [34–38]. Although the most adequate surgical method (i.e., MMS or WLE) for the treatment of DFSP remains controversial, there is evidence suggesting that MMS has lower rates of recurrence [23, 34, 39]. In some cases, DFSP might receive a simple excision because it is misdiagnosed as a benign mass, with high local recurrence (26–60%) [40]. FS-DFSP is highly aggressive and related to a high risk of local recurrence [13, 15]. FS changes can be commonly identified in primary tumors. In several studies, however, FS changes were detected only in recurrent tumors [12, 41]. Interestingly, our previous study showed that the proportion of FS-DFSP in the recurrent DFSP was higher than the primary DFSP [17]. In a multicenter study, Eva A et al. revealed that after WLE, patients with FS-DFSP more often experienced recurrence than those with C-DFSP [14]. In our recent study, we found that after MMS, FS change was an independent prognostic factor for local recurrence in both univariable and multivariable analyses [14].

It has been reported that 92–100% of DFSP cases usually show diffuse CD34 staining, can be positive for vimentin, nestin and apolipoprotein D, and can be negative for cytokerins, smooth muscle actin smooth muscle actin, S100, CD56, factor XIIIa, Stromelysin 3 and cathepsin K [24, 25, 42]. CD34 is reported to be negative in up to 50% of DFSP in FS-DFSP [43]. In the current study, the negative ratio of CD34 in FS-DFSP was significantly lower than that in C-DFSP. Sasaki indicated that the Ki-67 index in FS-DFSP is significantly higher than the Ki-67 index in C-DFSP (C-DFSP: 8.9% vs FS-DFSP: 21.5%) [44]. Similarly, in our study, the average Ki-67 index in FS-DFSP cases was significantly higher than that in C-DFSP cases (C-DFSP: 8.1% vs FS-DFSP: 18.1%). As a nuclear protein, Ki-67 is related to ribosomal RNA synthesis and has an essential function in cell proliferation. Khor et al. indicated that a high index of Ki-67 in prostate cancer was related to an increased risk of distant metastasis, cancer-specific mortality and overall death [45]. Several studies have shown that high Ki-67 levels were correlated with an obviously worse overall survival rate in mantle-cell lymphoma [46, 47]

The BP neural network is a kind of multilayer feedforward network that uses the error back-propagation algorithm. The BP neural network was first proposed by Paul Werbos in 1974, but it has not been widely recognized. In the 1980s, Rumelhar et al. renamed the BP algorithm, which was included in "Parallel Distributed Processing" [48–51]. Recently, the BP algorithm became the most widely used algorithm in neural networks. It was reported that approximately 90% of neural networks were based on the BP algorithm [19, 20]. At present, the BP neural network is widely used in disease recognition and diagnosis [19, 20]. In the present study, when the number of invisible layers is 10, the Levenberg–Marquardt algorithm can complete the learning of the entire training set sample size in 31 runs. The correct rates of classification and misdiagnosis were 84.1% and 15.9%, respectively. The classification accuracy and feasibility of the BP neural network model are high in FS-DFSP.

The retrospective nature of this research is the main limitation of the current study. In addition, long-term follow-up data were lacking in the current study. Nonetheless, this is one of the largest studies of DFSP, and despite its limitations, our study can provide valuable information to aid in clinical practice.

## Conclusion

Although the clinical characteristics of FS-DFSP might resemble those of C-DFSP, FS-DFSP usually occurs in older patients. In contrast to that in C-DFSP, the expression of CD34 in FS-DFSP tumor tissues is negative. The Ki-67 index in FS-DFSP is significantly higher than the Ki-67 index in C-DFSP. The BP neural network model constructed by the Levenberg–Marquardt algorithm has a high classification accuracy and feasibility for FS-DFSP and may be used as a method for clinical auxiliary identification.

## Data Availability

The datasets used and/or analyzed during the current study available from the corresponding author on reasonable request.

## Abbreviations

DFSP: Dermatofibrosarcoma protuberans
C-DFSP: Conventional dermatofibrosarcoma protuberans
FS-DFSP: Fibrosarcomatous dermatofibrosarcoma protuberans
ANN: An artificial neural network
BP: Back-propagation
